# Severe trauma associated cardiac failure

**DOI:** 10.1186/s13049-024-01175-4

**Published:** 2024-01-22

**Authors:** Maximilian Dietrich, Frank Weilbacher, Stephan Katzenschlager, Markus A. Weigand, Erik Popp

**Affiliations:** Medical Faculty Heidelberg, Department of Anesthesiology, Heidelberg University, Im Neuenheimer Feld 420, 69120 Heidelberg, Germany

**Keywords:** Traumatic cardiac arrest (TCA), Cardiac failure, Transfusion-associated cardiac overload (TACO), Advanced trauma care, Severe Trauma associated Cardiac Failure (STAC)

## Abstract

Although significant efforts have been made to enhance trauma care, the mortality rate for traumatic cardiac arrest (TCA) remains exceedingly high. Therefore, our institution has implemented special measures to optimize the treatment of major trauma patients. These measures include a prehospital Medical Intervention Car (MIC) and a ‘code red’ protocol in the trauma resuscitation room for patients with TCA or shock. These measures enable the early treatment of reversible causes of TCA and have resulted in a significant number of patients achieving adequate ROSC. However, a significant proportion of these patients still die due to circulatory failure shortly after. Our observations from patients who underwent clamshell thoracotomy or received echocardiographic evaluation in conjunction with current scientific findings led us to conclude that dysfunction of the heart itself may be the cause. Therefore, we propose discussing severe trauma-associated cardiac failure (STAC) as a new entity to facilitate scientific research and the development of specific treatment strategies, with the aim of improving the outcome of severe trauma.

Trauma claims the lives of 4.4 million people worldwide each year, accounting for nearly 8% of all deaths [1]. Despite major efforts to improve trauma care, the mortality rate from traumatic cardiac arrest (TCA) remains extremely high. However, younger patients with a low burden of chronic disease are particularly affected. If resuscitation is successful, neurological outcome has been reported to be much better than for other causes of cardiac arrest [2].

The mechanisms and causes of TCA differ significantly from those of a medical context. Mostly hypovolemic, obstructive, and neurogenic shock needs to be treated immediately to enable return of spontaneous circulation (ROSC) which is represented in a special treatment algorithm for TCA in the current European Resuscitation Council (ERC) guidelines [2]. To achieve this, well-trained pre-hospital teams with subsequent specialized in-hospital trauma care are needed to increase the chances of survival.

In Germany, a two-tier response system is in place. To any out-of-hospital cardiac arrest, a physician response unit and an ambulance are dispatched. Additionally, our institution implemented a Medical Intervention Car (MIC) at the University Hospital of Heidelberg to optimize the treatment of major trauma patients, including TCA. The MIC is staffed by an experienced emergency medical team of at least two doctors. Advanced prehospital interventions in major trauma include blood gas analysis, blood transfusion, transesophageal echocardiography, cerebral near-infrared spectroscopy, resuscitative endovascular balloon occlusion of the aorta (REBOA), and clamshell thoracotomy with manual aortic compression [3].

Further, a “code red” protocol was installed in the trauma resuscitation room at Heidelberg University Hospital for patients with TCA [4]. With these measures, we can treat reversible causes of TCA early and achieve adequate ROSC in a significant number of patients. However, a considerable proportion of these patients still die of circulatory failure after a short time, even though reversible causes such as exsanguination, pericardial tamponade, tension pneumothorax, and spinal cord injury could be adequately treated or ruled out as causes of recurrent circulatory failure.

Our observations from patients who underwent clamshell thoracotomy or received echocardiographic evaluation led us to conclude that dysfunction of the heart itself could be the reason for it. These patients had a physiological heart rhythm, no direct trauma to the myocardium, and no known pre-existing heart disease. They were unable to maintain circulation over a prolonged period despite adequate volume status. We therefore propose to introduce severe trauma-associated cardiac failure (STAC) as a new entity to enable scientific research and the development of specific treatment strategies. We believe it is crucial to describe STAC and thereby raise awareness of the problem, as the normal response of clinicians to hypotension in trauma patients is to administer fluids. In STAC patients, this could be detrimental as cardiac overload exacerbates the problem. Recent reports showed that elevated troponin serum concentrations were more frequently seen in non-survivors after TCA [5]. This corroborates our hypothesis that a combined pathomechanism consisting of myocardial hypoxia in the phase of severe trauma, transfusion-associated cardiac overload (TACO), inflammatory response, neurogenic stunned myocardium, sympathetic storm, and ischemic reperfusion damage including factors like electrolyte disturbances, hyperlactatemia, and cytokines is responsible for STAC [6, 7]. In non-traumatic cardiac arrest, myocardial ischemia and reperfusion, stunning, and hibernation of the myocardium are well-known phenomena [8, 9]. So far, this has not been shown in TCA or traumatic shock causes and clinical data on cardiac function following major trauma are scarce [10]. However, recent data from animal models show that severe trauma leads to decreased myocardial contractility [11]. In trauma patients with persisting shock or cardiac arrest despite sufficient treatment of reversible obstructive, hypovolemic, and neurogenic causes of TCA in the initial phase, a cardiogenic shock component should be considered irrespective of the absence of preexisting cardiac diseases. Thinking outside the box and using strategies rarely used in trauma patients, such as transesophageal echocardiography, inotropic therapy, or veno-arterial extracorporeal membrane oxygenation (ECMO), could potentially be lifesaving in STAC patients. To gain a better understanding of the incidence of STAC, prospective studies with continuous echocardiographic evaluation and biomarker monitoring during resuscitation of post-ROSC TCA are needed [12]. A possible definition could be based on echocardiographic findings and hemodynamic stability, including the need for vasoactive medication. In addition, clinical trials should include TCA patients with ROSC, as they have been excluded to date [13]. To elucidate the underlying pathomechanism further research in adequate animal models should be performed. Already established registries can be utilized for recruitment and data collection.

We believe that STAC should be reported to draw attention to it and allow further investigation and recognition during trauma resuscitation, which is crucial to avoid harmful fluid overload and initiate appropriate therapy for the cardiogenic shock component. To enhance the recognition of STAC in the early in-hospital phase, we are changing our standard operating procedure to make transesophageal echocardiography standard practice in the trauma room for all “code red” patients with shock, ROSC, or ongoing TCA.

## Data Availability

Not applicable.

## Abbreviations

ECMO: Extracorporeal membrane oxygenation
ERC: European Resuscitation Council
MIC: Medical Intervention Car
REBOA: Resuscitative endovascular balloon occlusion of the aorta
ROSC: Return of spontaneous circulation
STAC: Severe trauma-associated cardiac failure
TACO: Transfusion-associated cardiac overload
TCA: Traumatic cardiac arrest
